# Investigating a psychological model of mental conditions and coping during the COVID-19 pandemic driven by participatory methods

**DOI:** 10.1007/s00127-022-02316-9

**Published:** 2022-06-21

**Authors:** S. K. Simblett, S. Jilka, S. Vitoratou, C. Hayes, D. Morris, E. Wilson, C. Odoi, M. Mutepua, J. Evans, E. Negbenose, S. M. Jansli, G. Hudson, A. Adanijo, E. Dawe-Lane, V. Pinfold, T. Wykes

**Affiliations:** 1 Psychology Department, King’s College London, London, UK; 2 Department of Biostatistics and Health Informatics, King’s College London, London, UK; 3 Maudsley Biomedical Research Centre, South London and Maudsley NHS Foundation Trust, London, UK; 4 The McPin Foundation, London, UK

**Keywords:** Coping, COVID-19, Mental health, Participatory methods

## Abstract

**Background:**

There is evidence of increased mental health problems during the early stages of the COVID-19 pandemic. We aimed to identify the factors that put certain groups of people at greater risk of mental health problems.

**Methods:**

We took a participatory approach, involving people with lived experience of mental health problems and/or carers, to generate a set of risk factors and potential moderators of the effects of COVID on mental health. An online cross-sectional survey was completed by 1464 United Kingdom residents between 24th April and 27th June 2020. The survey had questions on whether respondents were existing mental health service users and or carers, level of depression (PHQ9) and anxiety (GAD7), demographics, threat and coping appraisals, perceived resilience (BRS), and specific coping behaviours (validated as part of this study). The relationship between responses and coping strategies was measured using tetrachoric correlations. Structural equation modelling was used to test the model.

**Results:**

A model significantly fit our data (rel χ^2^ = 2.05, RMSEA = 0.029 95%, CI (0.016, 0.042), CFI = 0.99, TLI = 0.98, SRMR = 0.014). Age and coping appraisal predicted anxiety and depression. Whereas, threat appraisal and ethnicity only predicted anxiety, and resilience only predicted depression. Additionally, specific coping behaviours predicted anxiety and depression, with overlap on distraction.

**Conclusions:**

Some, but not all, risk factors significantly predict anxiety and depression. While there is a relationship between anxiety and depression, different factors may put people at greater risk of one or the other during the pandemic.

## Introduction

The United Kingdom (UK) Government introduced a national lockdown to reduce the COVID-19 transmission rates, which included social distancing regulations and quarantining measures. Emerging research has shown a modest increase in mental health problems, including depression and anxiety, in the UK during the early stages of the pandemic [14, 28, 29]. While the prevalence of mental health problems reduced when lockdown restrictions were lifted in May 2020, there have been subsequent increases in depression and anxiety in response to later lockdowns [14]. Research has highlighted several aspects of an individual’s life (e.g., living with children, living in an urban area, household size, being unemployed, or a lower household income) that present an additional risk to the deleterious mental health effects of the pandemic [14, 28, 29].

Many factors have been proposed to moderate poor mental health during pandemics and periods of quarantine [7] with some recent indication that certain groups, such as people with pre-existing mental health problems and caregivers, may be at greater risk [22]. Understanding why this might be the case is important for preventing problems in the future. Although numerous studies have examined the mental health burden of the pandemic for adults and children, few have explored the impact depending on whether people are service users and carers, in addition to the interplay between situational appraisal, coping behaviours, resilience, and mental health outcomes during the pandemic. We took an approach grounded in participatory methods and conducted semi-structured interviews with people who had experience of mental health problems and or identified as carers, asking about their experience of coping in the early stages of the COVID-19 pandemic (during the first lockdown period) [35]. A series of themes emerged that have driven us to develop and test a psychological model of mental ill health and coping during the COVID-19 pandemic.

The following factors emerged from our participatory approach [35].

1. *Key appraisals of the situation* Two main appraisals emerged: (1) the perceived severity of the virus and the fear associated with the risk of death; and (2) their perceived ability to cope. Existing psychological models emphasise that if people’s estimation of danger or threat exceeds their estimation of coping, then levels of anxiety increase [17]. Emerging quantitative analyses during the COVID-19 pandemic demonstrate a significant role of threat and coping appraisals in moderating behaviour [1], which may extend to mood and anxiety.
2. *Specific coping behaviours* Table 1 lists the most common themes, likely to either exacerbate mental health problems or be protective. The themes span the breadth of coping styles proposed in the previous research: problem focussed or emotional focussed, approach, and avoidance [13, 16], but are specific to coping with the COVID-19 pandemic.
3. *Further ‘at risk’ groups* The potential differential impact of the COVID-19 pandemic on people of different ages, genders, and from an ethnic minority group (excluding white minorities) was raised, and preliminary findings support these concerns [32, 33].
4. *Perceived resilience* High levels of perceived resilience or the ability to bounce back from adversity was thought to be a protective factor for mental health problems. This is supported by COVID-19-specific research [23].
5. *Time* Mental health service users and carers, although interviewed at a single time point, reported difficulties looking forward, with concerns that their mental health would deteriorate the longer they are required to cope with the measures introduced to reduce the spread of the virus. There is no evidence to support signs of mental health deterioration (e.g., increased suicidal ideation) and the evidence for change in any direction is sparse.

**Table 1:** Percentages of endorsed coping items in order of most frequent use

Full item	Item label	*n*	% Yes	% Agreement	Kappa (95% CI)
Following government recommended behaviours (e.g., washing hands more and keeping social distance)	Recommended behaviours	1290	97.6	96.8	*
Communicating more using technology	Technology	1164	87.8	96.8	0.8 (0.4,1)
Trying to create a positive environment	Positive environment	1103	83.5	90.3	0.8 (0.5,1)
Taking things day-by-day	Day-by-day	1028	77.9	83.3	0.5 (0.1,0.9)
Trying to keep healthy (e.g., eating, sleeping well)	Healthy activities	1030	77.7	87.1	0.7 (0.4,1)
Exercising (e.g., going for a walk)	Exercise	1027	77.5	83.9	0.6 (0.3,0.9)
Keeping busy and doing things to distract oneself	Distract oneself	996	75.2	83.9	0.6 (0.3,0.9)
Trying to help others	Help others	979	73.9	87.1	0.7 (0.4,1)
Keeping essential appointments where possible	Attend appointments	871	66.5	90.3	0.4 ( – 0.2,0.9)
Finding ways to relax	Ways to relax	845	64.0	71.0	0.4 (0.1,0.7)
Checking the news or social media too frequently	Check media	815	61.6	80.6	0.6 (0.3,0.9)
Having too much time to think	Time to think	774	60.0	83.9	0.7 (0.4,0.9)
Keeping a to-do list	To-do-lists	791	59.7	80.6	0.4 (0,0.8)
Speaking openly about problems with someone	Speak openly	767	58.1	80.6	0.5 (0.1,0.8)
Planning one’s day	Plan day	711	53.9	80.6	0.6 (0.3,0.9)
Sticking to a routine	Sticking to routine	671	50.8	83.9	0.7 (0.4,0.9)
Learning something new, starting a project or mastering an existing skill	Learning	642	48.6	83.3	0.7 (0.4,0.9)
Spending more time in bed	More time in bed	599	45.2	80.6	0.6 (0.3,0.9)
Making time for positive self-reflection	Positive self-reflection	559	43.6	80.6	0.6 (0.3,0.9)
Using alcohol, drugs, excessive exercise of food	Using substance	538	40.7	74.2	0.4 (0.1,0.7)
Using mindfulness or meditation techniques	Mindfulness	501	37.8	90.3	0.8 (0.6,1)
Watching TV or films excessively to fill the time	Watch media	492	37.1	80.6	0.6 (0.3,0.9)
Spending time thinking about what would happen if he/she became physically unwell	Physical health	447	34.7	77.4	0.5 (0.2,0.8)
Stockpiling things I need	Stockpile things	418	31.7	93.5	0.8 (0.6,1)
Talking to people more	Talk to people	410	30.9	74.2	0.3 (0,0.7)
Using health and wellness apps	Health apps	376	28.5	90.3	0.8 (0.6,1)
Feeling able to make plans	Able-to-plan	350	27.2	77.4	0.6 (0.3,0.8)
Spending more money than usual	Spend money	291	22.0	83.3	0.6 (0.3,0.9)
Turning to religion and spirituality a source of support	Spirituality	221	16.7	96.8	0.9 (0.7,1)

*Kappa could not be computed as all individuals selected the same option in time 2

There is no, single, model that explores all these variables. Our hypotheses have been driven by participatory methods and we have used a path analysis to help quantify the relationship between the following factors: whether people were mental health service users and or carers, current mental health problems (depression and anxiety, separately), demographic characteristics (most notably, age and ethnicity), threat and coping appraisals, perceived resilience, and specific coping behaviours generated by mental health service users and carers in our previous interviews. The aim was to validate the psychometric properties of a COVID-19 coping strategies measure for mental health service users and to then identify what collection of factors put people at greater risk of mental health problems, and to identify potential avenues for future investigation of interventions that will improve people’s experiences during pandemics.

## Methods

### Design

This was a cross-sectional study using Qualtrics, an online survey software and snowball sampling.

### Participants and recruitment

We recruited two samples of participants through social media and other online channels. Sample 1 was used to test a psychological model of mental ill health and coping during the COVID-19 pandemic. Sample 2 was gathered to check the reliability and validity of a coping strategy measure developed for this study. To be included in either sample, participants had to be from the UK and aged at least 16 years. We did not limit respondents based on any other characteristics but purposely sought responses from people belonging to pre-identified minority groups (e.g., men and non-white people) [37] through specific social media adverts and support from charities. For sample 1, respondents were categorised based on whether or not they were using mental health services at the time of survey completion. All participants were also asked whether they identified as a carer of someone with a mental health condition. For sample 2, it consisted of people who were mental health service users [36].

### Procedure

The whole study was approved by King’s College London ethics committee (sample 1: HR-19/20–18,180 and sample 2: HR/DP-20/21–21,830). All participants were provided with the study information sheet when they opened the survey link and consented before answering any questions. For sample 1, the survey was published online on 24th April 2020 and data collection stopped on 27th June 2020. For sample 2, data were collected between 15th of February and 18th of June 2021.

### Our participatory approach

All parts of the research, including the survey design, data collection, and interpretation of the findings, were conducted by researchers with experience of mental health problems. In this way, we continued to use a participatory approach throughout [35].

### Measures

Our survey used to gather information in sample 1 was generated from the discussions with service users and carers, who also provided feedback on the final survey version as part of our patient involvement strategy [35].

We collected the following outcome measures:

1. Mental health service user and carer status.
2. Demographics: age, gender, and ethnicity [grouped into ethnic minority groups (excluding white minorities) or White].
3. Clinical scales:
  – Brief Resilience Scale (BRS) [37]: a 6-item self-report measure assessing an individual’s beliefs about the ease with which they recover from stressful events. Higher scores indicate a higher appraisal of one’s own resilience. Mean resilience scores in the previous studies range from 3.53 to 3.98.
  – Patient Health Questionnaire (PHQ-9) [38]: a self-report measure with nine items corresponding to the diagnostic criteria for DSM-IV major depressive disorder. Scores of 5, 10, 15, and 20 represent cut-off points for mild, moderate, moderately severe, and severe depression, respectively.
  – Generalized Anxiety Disorder Assessment (GAD-7) [39]: a seven-item screening measure for generalized anxiety disorder, commonly co-morbid with depression. Scores of 5, 10, and 15 are taken as the cut-off points for mild, moderate, and severe anxiety, respectively.
4. Single-item measures:
  – Perceived ability to cope: Participants were asked ‘how confident do you feel that you can cope right now?’ on a scale of 0–10 with higher scores indicating better ability to cope.
  – Perceived severity of the virus: Participants were asked ‘How serious or dangerous do you think this virus is?’ on a scale of 0–10 with higher scores indicating higher perceived severity.
  – Coping strategies: 29 specific items describing different ways of coping, as outlined in Table 1, generated by discussions with mental health service users and carers.
5. Time: number of days since the start of lockdown (23rd March 2020) was used as a marker of time, which had been suggested by service users as important.

For sample 2, we gathered demographics, PHQ-9 and GAD-7 scores, alongside endorsements of the list of 29 coping strategies. In addition, we collected responses to a validated measure of coping style:

–  Brief COPE [8]: a 28 item self-report questionnaire designed to measure helpful and unhelpful ways to cope with a stressful life event. Each item is scored from ‘I haven’t been doing this at all’ (1) to ‘I’ve been doing this a lot’ (4).

### Data analysis

Summary data were calculated for GAD-7, PHQ-9, and BRS scores. Missing data were pro-rated using the mean score where participants had completed at least 5/7 items for GAD-7, 7/9 for PHQ-9, and 4/6 for the BRS following the procedure of Arrieta et al. [2]. Biserial correlations were used to explore the associations between binary items (e.g., the coping strategies) and continuous scores (e.g., the PHQ-9 and GAD-7). Test–retest reliability (stability) of the coping strategies measure was evaluated using Cohen’s Kappa coefficient [9] for binary items. For interval data, two measures of agreement were used namely the Psi non-parametric concordance coefficient [24] and the intraclass correlation coefficient [34].

To measure the relationship between binary responses to coping strategies, tetrachoric correlations were computed. Structural equation modelling was used to construct formative models [11, 12] for two outcomes, depression measured using PHQ9 and anxiety measured using GAD7. The fit of the SEM models was evaluated using absolute and comparative fit indices, namely the relative Chi-square (rel χ2: values close to 2 suggest close fit; [19], Root Mean Square Error of Approximation [RMSEA—values below 0.05 indicate close fit; [20]], Comparative Fit Index [CFI: values above 0.95 indicate a close fit; [4]], Taylor–Lewis Index [TLI: values above 0.95 are required for close fit; [20]], and Standardized Root Mean Residual [SRMR: values below 0.05 suggest a close fit; [20]]. All latent variable analyses were conducted in MPlus software [26].

## Results

### Participant characteristics

We recruited 1464 participants who were on average 41.27 (SD = 14.51) years old, mostly women (78%), and White (76%). 19.5% identified as a mental health service user and 13.8% identified as a carer. The sample reported lower-than-average perceived resilience (3.01, SD = 0.94; range 1–5), mild depression (9.42, SD = 7.03; range 0–27), and mild anxiety (8.15, SD = 5.98; range 1–21). On average, participants scored 6.24 (SD = 2.48) out of 10 for their perceived ability to cope, and 7.80 (SD = 2.00) out of 10 for their perceived level of virus severity. The percentages of endorsed coping behaviour items in order of most frequent use can be found in Table 1. The top three strategies were (1) following government recommended behaviours (e.g., washing hands more and keeping social distance), (2) communicating more using technology, and (3) trying to create a positive environment.

## Psychometric properties of the COVID-19 coping strategies measure

### Stability

The coping strategies measure had satisfactory stability (agreement between timepoints was at least 70% with most more than 80%). Kappa has higher than 0.5 in 23 of 28 strategies, with only ‘talk to people’ having a low coefficient of 0.3. See Table 1 for a summary of these statistics.

### Validity

Table 2 presents the biserial correlations between the endorsement of each copying strategy and the factor and total scores of already validated Brief COPE measure. For the majority of the strategies, there was no association, but for those which were significant, the correlation was strong (0.4–0.6) and highly significant (*p* < 0.001). For almost half of the coping strategies, there was a positive correlation with the total number of strategies endorsed, indicating that these behaviours were endorse by those who tend to endorse multiple coping strategies.

**Table 2:** Biserial correlations to establish validity of the COVID-19 coping strategies measure

	Brief COPE total	Brief COPE problem-focussed	Brief COPE emotion-focussed	Brief COPE avoidance-focussed	GAD-7 time point 1	PHQ-9 time point 1	Number of strategies endorsed
Brief COPE problem-focussed	0.718**						
Brief COPE emotion-focussed	0.878**	0.477**					
Brief COPE avoidance-focussed	0.513**	– 0.063	0.334*				
GAD-7 time point 1	0.353*	c0.062	0.338*	0.561**			
PHQ-9 time point 1	0.185	– 0.302*	0.27	0.538**	0.700**		
Number of strategies endorsed	– 0.616**	– 0.498**	– 0.560**	– 0.214	– 0.215	– 0.021	1
Talk to people	– 0.189	– 0.199	– 0.252	0.108	0.024	– 0.005	0.309*
Help others	– 0.088	– 0.027	– 0.124	– 0.028	– 0.096	– 0.073	0.186
Spirituality	– 0.031	– 0.189	– 0.115	0.327*	0.366*	0.354*	0.130
To do lists	– 0.142	– 0.118	– 0.261	0.138	– 0.152	– 0.016	0.467**
Speak openly	– 0.517**	– 0.402**	– 0.524**	– 0.125	– 0.277	– 0.109	0.506**
Mindfulness	– 0.461**	– 0.381**	– 0.494**	– 0.045	– 0.093	0.038	0.617**
Communicating technology	– 0.177	0.033	– 0.247	– 0.176	– 0.026	– 0.021	0.127
Positive environment	– 0.535**	– 0.554**	– 0.547**	0.058	– 0.008	0.107	0.679**
Using substances	– 0.456**	– 0.075	– 0.430**	– 0.530**	– 0.359*	– 0.360*	0.248
Healthy activities	– 0.172	– 0.259	– 0.205	0.167	0.078	0.141	0.476**
Distracting oneself	– 0.197	– 0.101	– 0.186	– 0.138	– 0.121	– 0.036	0.408**
Sticking to routine	– 0.004	– 0.132	– 0.005	0.173	0.199	0.338*	0.287
Exercise	0.124	– 0.221	0.201	0.347*	0.314*	0.462**	0.23
More time in bed	– 0.290	0.093	– 0.295*	– 0.492**	– 0.365*	– 0.346*	0.218
Watch media	– 0.017	0.347*	– 0.061	– 0.422**	– 0.363*	– 0.409**	0.019
Plan day	– 0.059	– 0.310*	0.068	0.158	– 0.055	0.185	0.371*
Check media	– 0.089	0.093	– 0.122	– 0.192	– 0.441**	– 0.381*	0.195
Health wellness apps	– 0.562**	– 0.466**	– 0.481**	– 0.223	– 0.089	– 0.006	0.669**
Spend money	– 0.177	0.017	– 0.187	– 0.239	– 0.307*	– 0.353*	0.337*
Learning	– 0.131	– 0.340*	– 0.023	0.13	0.202	0.148	0.344*
Day by day	– 0.106	– 0.141	0.007	– 0.108	– 0.024	0.163	0.158
Recommended behaviours	– 0.116	– 0.147	– 0.081	– 0.003	0.074	0.191	0.006
Attending appointments	– 0.055	0.011	0.018	– 0.187	– 0.187	– 0.150	– 0.068
Finding ways to relax	0.002	– 0.115	0.027	0.121	0.349*	0.262	0.102
Stockpiling things	0.029	– 0.019	0.071	0.003	0.032	0.066	0.226
Time to think	– 0.068	0.228	– 0.049	– 0.417**	– 0.440**	– 0.426**	0.077
Positive self reflection	– 0.111	– 0.264	– 0.094	0.182	0.232	0.196	0.393**
Worrying physical health	– 0.232	– 0.11	– 0.077	– 0.372*	– 0.490**	– 0.305*	0.312*
Able to make plans	– 0.365*	– 0.491**	– 0.290	0.074	0.281	0.326*	0.287

## Psychological model of mental ill health and coping

### Correlations

The item intercorrelations varied from not significant to low or moderately low (0 to 0.5 on absolute value, average intercorrelation = 0.05). The low item-inter correlations typically lead to omitting a large proportion of the indicators and result in less than satisfactory internal consistency if common factor analysis models are fitted. These statistical results are indicative of formative (causal) indicators, rather than reflective ones used in factor analysis models. We therefore proceeded by constructing a formative model for each outcome (PHQ9 or GAD7 scores) using SEM methodology described below.

### Structural equation modelling

The first step was to identify which indicators (coping behaviours) affected the two outcomes of interest (depression and anxiety). For each outcome we fitted a series of multiple regression models using a stepwise forward procedure with predictor variables including age, gender, ethnicity, whether a person is a current ‘service user’ or not, whether a person is a carer or not, days since lockdown, BRS, the ability to cope score, the virus severity appraisal, and the other outcome.

**Fig. 1:**
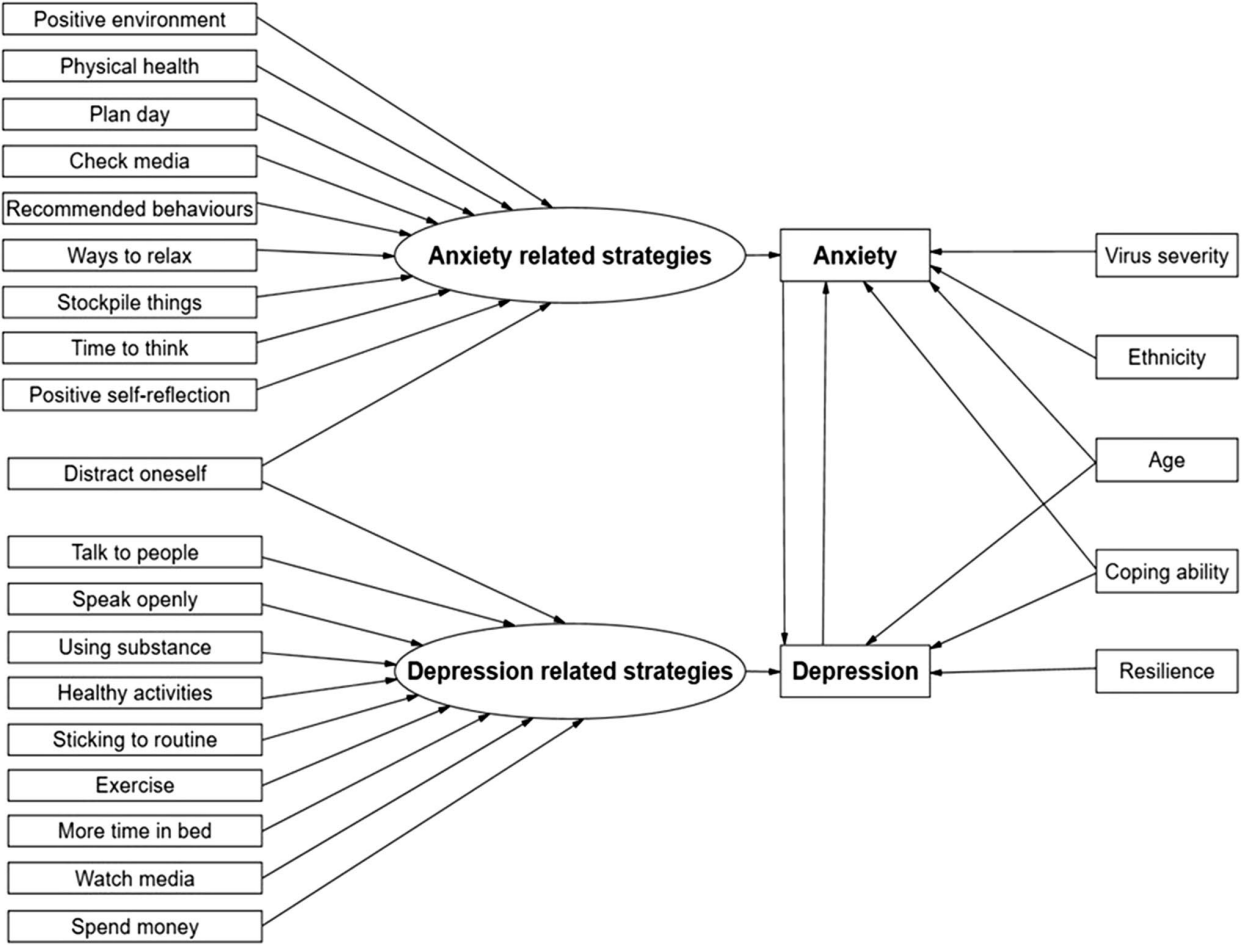
A structural equation model of coping strategies contributing to experiences of anxiety and depression during the COVID-19 pandemic

Current mental health service use and carer status were not significant contributors and were, therefore, not included in the final model. For anxiety, the final model included significant effects for age, ethnicity, ability to cope, virus severity appraisal, and depression. For depression on the other hand, the significant predictors were age, ability to cope score, perceived resilience, and anxiety. The second step was to add to the model a formative latent factor constructed by all binary coping behaviour items and gradually delete those that were not significant (using a stepwise backwards procedure). The resulting formative factors related to anxiety and depression were constructed by different sets of indicators, with only one item being present at both (e.g., “I have been keeping busy and doing things to distract myself”). Finally, the two models were merged in one SEM model where the two outcomes can correlate in addition to their relationship with their predictors. The final SEM model with the corresponding coefficients is presented in Fig. 1. All coefficients were statistically significant, and the model had close fit to our data (rel χ^2^ = 2.05, RMSEA = 0.029 95% CI (0.016, 0.042), CFI = 0.99, TLI = 0.98, SRMR = 0.014). Statistics relating to the contributions of each individual variable are presented in Table 3.

**Table 3:** Statistical contributions for individual variables included in the model

Latent construct	Label	Estimate	S.E	*P* value
Formative anxiety indicators	Positive environment	0.71	0.19	< 0.001
	Distract oneself	0.35	0.16	0.030
	Plan day	0.35	0.15	0.016
	Check media	0.69	0.13	< 0.001
	Recommended behaviours	– 0.99	0.42	0.018
	Ways to relax	– 0.62	0.15	< 0.001
	Stockpile things	0.29	0.14	0.038
	Time to think	0.68	0.14	< 0.001
	Positive self-reflection	– 0.47	0.14	0.001
	Physical health	1.14	0.13	< 0.001
Formative depression indicators	Talk to people	0.25	0.11	0.017
	Speak openly	0.30	0.10	0.003
	Using substance	– 0.79	0.10	< 0.001
	Healthy activities	0.50	0.13	< 0.001
	Distract oneself	0.29	0.12	0.015
	Sticking to routine	0.28	0.10	0.007
	Exercising	0.47	0.12	< 0.001
	More time in bed	– 0.67	0.10	< 0.001
	Watch media	– 0.36	0.11	0.001
	Spend money	– 0.46	0.12	< 0.001
Predictors of GAD7	Age	– 0.02	0.01	0.002
	Coping ability	– 0.74	0.07	< 0.001
	Virus severity	0.11	0.05	0.023
	Depression	0.34	0.04	< 0.001
	Ethnicity	– 0.62	0.30	0.039
	Formative anxiety	1.45	0.12	< 0.001
Predictors of PHQ9	Age	– 0.03	0.01	0.001
	Coping ability	– 0.68	0.08	< 0.001
	Anxiety	0.32	0.05	< 0.001
	Resilience	– 1.24	0.15	< 0.001
	Formative depression	– 2.25	0.14	< 0.001

## Discussion

The COVID-19 coping strategies measure was found to have sound psychometric properties, both in terms of stability and construct validity. This allowed us to explore a psychological model of mental ill health and coping. The final model allows us to identify variables that seem to relate to current anxiety and depression, and many validate the suggestions of our service user advisory groups. Our findings support mental health service users concerns that how the individual perceives the severity of the virus may contribute to levels of distress. Threat appraisal biases have long been a target for understanding anxiety [6] and specifically in the context of pandemics [41]. Our additional finding that ethnic minorities (excluding white minorities) were at greater risk of mental health problems early during the COVID-19 pandemic is also supported within the existing literature [32]. However, this was specific to anxiety. While disparities may be multifactorial, research has shown that mortality from COVID-19 was much higher for people from ethnic minority groups (excluding white minority) [30]. Therefore, it is logical to assume a relationship between ethnicity and perceived severity of the virus and this may help to understand why ethnicity is a specific predictor of anxiety. We have identified a series of specific coping behaviours that may have put people at greater risk of anxiety, including frequent checking behaviours, stockpiling, having too much time to think, worry about becoming physically unwell, not finding ways to relax or plan the day ahead, which are supported by previous findings [10, 21]. Interestingly, trying to create a positive environment also predicted anxiety; this may represent a problem-solving coping strategy that people employ to create a greater sense of control and manage the psychological impacts of social distancing, self-isolation, and quarantine.

In terms of risk of depression, specific coping behaviours were mostly different from the risk of anxiety with an emphasis on things such as substance misuse, not speaking openly to others, disrupted routine, excessive TV watching or escapism, spending time in bed, etc. Some of these areas have been identified as risk factors for depression [5, 31] and associated with psychological wellbeing in the context of the COVID-19 pandemic [18] but they have rarely, if ever, all been included in the same model. Similarly, this combination of specific coping behaviours has not been merged with an understanding of the contribution of resilience. In our findings, low resilience predicted higher levels of depression (but not higher levels of anxiety). This reflects the literature with more written on the preventative role of resilience in the development of depression [43] with the general conclusion being that recovery from stress or the ability to ‘bounce back’ keeps people well even if they have experienced mental health problems in the past.

There were several variables in our model that could be considered as ‘transdiagnostic’ predictors of anxiety and depression. Both age and coping appraisal have been widely studied in the context of pandemics and the impact on mental health [33, 41, 42] with younger people and those with fewer coping resources more likely to suffer distress. Only one specific coping behaviour contributed to predicting both anxiety and depression, that is, not keeping busy and doing things puts people at risk of both anxiety and depression. These replicates previous research, showing that distraction is a protective factor against depression (Response Style Theory) [27] and anxiety [3].

Some, but not all, risk factors identified by mental health service users significantly predict mental distress. First, being a current mental health service user or a carer did not put people at greater risk of anxiety or depression. These factors are not associated with poorer mental health and the variance is better explained by factors such as perceived resilience and coping resources. Second, some frequently endorsed coping behaviours, e.g., use of technology to communicate, did not significantly contribute to an increase in anxiety or depression. This is counter to hypotheses which suggest that adapted social interactions using technology are detrimental to mental health [25]. Third, while service users in our interviews [35] raised concerns that mental health would deteriorate the longer people were required to cope, time was not a predictor of either anxiety or depression in our model. Each effect was evaluated here in the presence of all other effects (controlled, adjusted) based on the full model in Fig. 1, and differences with previous research can be explained as these results considered one or a few effects at a time. The general psychological model of mental ill health and coping during the pandemic presented here is unique in the literature in terms of combining anxiety and depression measures with corresponding formative indices of coping strategies and descriptive characteristics of the sample.

## Strengths and limitations

As far as the authors are aware, this is the first paper to investigate risk factors of anxiety and depression, driven by participatory methods with mental health service users. We have begun to explain poor mental health during the first lockdown period of the COVID-19 pandemic, teasing apart specific and transdiagnostic reasons for anxiety and depression. Responses to our survey were anonymous, which may have avoided social desirability bias that can occur when responding to sensitive questions face-to-face [40]. The recruitment and data collection were carried out remotely due to COVID-19 policy. This may have encouraged the participation of some people who would usually be hard to reach and facilitated access to a large and geographically dispersed population improving the representativeness of the sample. This is particularly as we attempted recruitment through several different routes. However, the use of online surveys can lead to the digital exclusion of people who do not have access to technology and areas of diversity were not equally represented in our sample, leading to a limitation in the conclusions that can be drawn. While we have demonstrated some role of ethnicity in moderating experiences of anxiety, the sample was predominantly female and white, and this may have affected the power to detect this and other effects. Further research is needed that explores other ethnic groups and has a greater representation of men. We acknowledge that this present study was cross-sectional, which means that we cannot determine whether coping behaviours found to be associated with depression and anxiety, preceded and or were exacerbated by the COVID-19 pandemic. However, other emerging evidence from longitudinal studies do suggest that ways of coping may have influenced mental health condition trajectories. In a large longitudinal UK-based study by Fluharty et al. [15], people with greater use of problem-focussed, avoidant, and supportive coping were found to display more symptoms of mental health conditions, while greater use of emotion-focussed coping was associated with fewer symptoms of mental health conditions.

## Conclusions

We present a psychological model of mental ill health and coping during the COVID-19 pandemic grounded in the experiences of mental health service users. As part of this work, we have identified several areas that could be targets for intervention. These interventions may be psychological in nature, focussing on the reframing of appraisals and encouraging or discouraging certain behaviours, but others may be based on societal inequalities, such as the actual need for more resources, or real risks (e.g., of mortality from the virus for certain groups) and the need to prevent transmission.
